# Mutant p53 in Cancer Progression and Targeted Therapies

**DOI:** 10.3389/fonc.2020.595187

**Published:** 2020-11-06

**Authors:** Gaoyang Zhu, Chaoyun Pan, Jin-Xin Bei, Bo Li, Chen Liang, Yang Xu, Xuemei Fu

**Affiliations:** ^1^ Postdoctoral Research Center, Shunde Hospital, Southern Medical University (The First People’s Hospital of Shunde), Foshan, China; ^2^ Department of Biochemistry and Molecular Biology, Zhongshan School of Medicine, Sun Yat-sen University, Guangzhou, China; ^3^ Department of Experimental Research, Sun Yat-sen University Cancer Center, State Key Laboratory of Oncology in South China, Collaborative Innovation Center for Cancer Medicine, Guangdong Key Laboratory of Nasopharyngeal Carcinoma Diagnosis and Therapy, Guangzhou, China; ^4^ Shenzhen International Institute for Biomedical Research, Shenzhen, China; ^5^ Department of Pediatrics, The Eighth Affiliated Hospital, Sun Yat-sen University, Shenzhen, China; ^6^ Division of Biological Sciences, University of California, San Diego, La Jolla, CA, United States

**Keywords:** mutant p53 protein, gain-of-function, targeted therapy, tumorigenesis, drug resistance

## Abstract

*TP53* is the most frequently mutated tumor suppressor gene in human cancer. The majority of mutations of p53 are missense mutations, leading to the expression of the full length p53 mutant proteins. Mutant p53 (Mutp53) proteins not only lose wild-type p53-dependent tumor suppressive functions, but also frequently acquire oncogenic gain-of-functions (GOF) that promote tumorigenesis. In this review, we summarize the recent advances in our understanding of the oncogenic GOF of mutp53 and the potential therapies targeting mutp53 in human cancers. In particular, we discuss the promising drugs that are currently under clinical trials as well as the emerging therapeutic strategies, including CRISPR/Cas9 based genome edition of mutant *TP53* allele, small peptide mediated restoration of wild-type p53 function, and immunotherapies that directly eliminate mutp53 expressing tumor cells.

## Introduction

Tumor suppressor p53 is the principal cellular responder to various stress signals such as oncogene activation, DNA damage, hypoxia, reactive oxygen species (ROS), etc. Upon activation, p53 induces numerous cellular responses including cell cycle arrest to restore genetic integrity, or apoptosis, senescence, or ferroptosis to eliminate unrecoverable cells. Therefore, p53 is considered the “Guardian of the genome” to prevent accumulation of oncogenic mutations that lead to malignant tumor (1, 2).

Mutations in *TP53* are found in over half of human cancers, thus is known as the most commonly mutated gene in human cancers (3, 4). Different from many other tumor suppressor genes which generally undergo deletion or truncation in cancer cells, mutations in *TP53* allele are predominantly missense mutations which give rise to a single amino acid substitution in the full-length mutant protein (5). p53 protein is composed of three functional domains including a transactivation and proline rich domain, a central DNA-binding domain (DBD), and an oligomerization domain (6). While mutations can occur spontaneously throughout the p53 gene, the majority of p53 missense mutations are located in the central DBD region of the p53 gene, which binds to its consensus DNA binding sites to regulate its target gene expression. These missense mutations are divided into two categories: DNA contact mutations such as R248Q and R273H that directly occur at the amino acids mediating p53-DNA interaction, and conformational mutations that indirectly disrupt p53-DNA interaction by inducing local (R249S and G245S) or global (R175H and R282W) conformational changes due to the reduced thermostability caused by these mutations (5). Nearly one third of all p53 mutations occur at these six “hotspot” mutational residues (3).

Mutations in the p53 gene can appear at either the initial-stage or the late-stage during tumorigenesis depending on the origin of cancer types, and strongly facilitate the onset or progression of cancers (7). Functionally, p53 mutants (mutp53) not only lead to the loss of wild-type p53 functions, but can also result in a dominant negative effect by forming hetero-tetramers with the remaining wild-type p53 expressed from the other wild-type allele. p53 mutations are usually followed by the loss of heterozygosity (LOH) at the remaining wild-type *TP53* allele, leading to the complete loss of wild-type p53 in late-stage tumors, and further confer these cancer cells a selective advantage during cancer development (8, 9). Most p53 missense mutants acquire oncogenic gain-of-function (GOF) activities. For example, conformational changes of mutp53 enable them to interact with many transcription factors such as p63, p73, NF-Y, Sp1, ETS1/2, NF-κB, ATM, and SMADs, altering the transcription, cell cycle, apoptosis and metabolism of cancer cells. This changes lead to increased genetic instability, cellular proliferation, metastasis and chemo-/radio-resistance (10). In addition, the new transcriptional targets acquired by mutp53 is another well established GOF mechanism for mutp53 to promote cancer progression (5). Therefore, to compete for survival in a nutrient-deprived and hypoxic environment, the human tumor cells are under stringent selection for the loss of wild-type p53 function and acquirement of p53 mutants that possess GOF to promote the survival of tumor cells.

In this review, we focus on the gain-of-functions of mutp53 in malignant tumor progression and the current strategies targeting mutp53 for personalized therapeutic treatments, aiming to provide insights into targeted treatment of human cancers with p53 mutation.

## Mutant p53 Facilitates Cancer Progression

### Induction of Genetic Instability

As the “Guardian of the genome”, the fundamental goal of WT p53 is to maintain genetic stability by preventing the passage of genetic mutations to daughter cells (1). While p53 null cells still retain certain levels of checkpoint and DNA repair capacities, cells harboring p53 mutant proteins showed a dramatic higher level of genomic instability such as interchromosomal translocations and aneuploidy, indicating the oncogenic GOF activity of p53 mutants (8, 11, 12). These variations largely contribute to genetic diversity that expedites malignant tumor development. Mechanistically, the common p53 mutants can disrupt the earliest stage of DNA double-stranded break (DSB) damage responses by interacting with the nuclease Mre11 to suppress the recruitment of Mre11/Rad50/NBS1 (MRN) complex to the site of DNA DSB damage, leading to inactivation of ATM, the key DNA DSB damage sensor, and the resultant G_2_/M checkpoint impairment (8). Mutp53 can also induce genomic abnormality by inactivating DNA replication process. For example, some mutp53 proteins activate cyclin A to promote the formation of DNA replication origin and the intra-S phase checkpoint kinase CHK1 to stabilize the replication forks, facilitating the duplication of aberrant genomic DNAs (13).

### Accelerating Proliferation

Accumulating evidence has revealed that mutp53 promotes the limitless replicative potential and insensitivity to anti-growth signals during the malignant transformation of a normal cell, which are two of the key “hallmarks of cancer” (14, 15). Mutp53 was reported to physically interact with the major cell cycle regulator nuclear transcription factor Y (NF-Y), and recruits either acetyltransferase p300 or the main effector of Hippo pathway, YAP, to activate NF-Y target genes including *cyclin A*, *cyclin B*, *cdk1* and *cdc25C* (16, 17). Mutant p53 and YAP have also been found to form another trimeric transcriptional complex with TEAD to induce the expression of circular RNA *circPVT1*, which activates proliferative genes such as *aurka* and *mki67* (18). Mutp53 also regulates the expression of MicroRNA *miR-27a*, which promotes a sustained EGF-induced ERK1/2 activation, thereby facilitating cellular proliferation and tumorigenesis (19). In addition, p53 mutants also target key chromatin regulators including methyltransferases MLL1 and MLL2 and acetyltransferase MOZ, leading to genome-wide increases of active histone modifications H3K4me3 and H3K9ac to enhance proliferation (20). In addition, the *TP53* R249S mutant, frequently detected in HBV positive human hepatocellular carcinoma (HCC), has a unique GOF in regulating proliferation and survival of HCC cells by promoting c-Myc-dependent rDNA transcription essential for ribosomal biogenesis (21).

### Modulating Metabolism

Cellular metabolism of glucose, lipid, and nucleotide are the fundamental basis for cell survival, which undergo dramatic changes during malignant transformation. Emerging evidence shows that mutp53 proteins contribute to various aspects of these processes (22, 23). Rapidly dividing tumor cells rely mainly on aerobic glycolysis to meet their high energy and biosynthetic demand, a phenomenon known as Warburg effect (24). Mutp53 has been shown to activate the small GTPase RhoA and its downstream effector ROCK, to promote GLUT1 translocation to the plasma membrane and thus enhance glucose uptake and glycolytic rate (25). Under conditions of energy stress, mutp53 preferentially binds to the AMPKα subunit and directly inhibits the metabolic functions of AMPK signaling, leading to increased aerobic glycolysis as well as lipid production (26). Notably, the roles of mutp53 in promoting lipid metabolism are partly mediated by the mevalonate pathway, which is responsible for *de novo* cholesterol synthesis and generation of many important nonsterol isoprenoid derivatives. Mutp53 is recruited to the promoters of several mevalonate pathway genes to induce their expression through its interaction with the master transcription factor SREBP1/SREBP2 (27). In addition, the mevalonate pathway-DNAJA1 axis as well as the STAT3-mevalonate pathway axis are both found to prevent mutp53 from being degraded by CHIP ubiquitin ligase, forming a positive-feedback loop to ensure rapid lipid synthesis (28, 29). Mutp53 proteins were also reported to promote nucleotide synthesis through its interaction with ETS2 to activate numerous nucleotide metabolism genes (*RRM2*, *dCK*, *TK1*, *GMPS*, *IMPDH1*, *PAICS*) involved in both the *de novo* and the salvage pathways required for nucleotide synthesis, leading to elevated nucleotide pools and the subsequent enhancement of GTP dependent protein (GTPase) activity (30, 31). Collectively, these findings highlighted the metabolic reprogramming roles of mutp53 in cancer cells.

### Promoting Metastasis

Metastasis is another “hallmark of cancer” and contributes to over 90% of cancer-associated deaths (15, 32). Epithelial-to-mesenchymal transition (EMT) is the first and the most essential step of metastasis that allows the cells to change their morphology to gain enhanced migration and invasion capacity. Mutp53 were reported to promote the expression of several key EMT-related transcription factors including ZEB1, SLUG, and TWIST1 through transcriptional, post-translational and epigenetic modifications, possibly in a cell type dependent manner (33–35). In endometrial cancer tissues, mutp53 represses the expression of *miR-130b*, which negatively regulates *ZEB1* (33). In non-small-cell lung cancers (NSCLCs), mutp53 inactivates MDM2 mediated SLUG degradation and result in high SLUG and low E-cadherin expression (34). While in prostate cells, mutp53 induces the reduction of H3K27me3 repression mark on TWIST1 promoter (35). Besides, several lines of evidence suggest that p63 is an effector of mutant p53 mediated metastasis. Mutp53 forms a ternary complex with p63 and phosphorylated Smad2 in the presence of TGF-β signaling, which repressed the activation of p63 downstream metastasis suppressor genes *Cyclin G2* and *Sharp-1* (36). Mutp53 also inhibits p63 mediated inactivation of Rab-coupling protein (RCP), resulting in enhanced α5β1-integrin and EGFR trafficking to the plasma membrane and the constitutive activation of EGFR/integrin signaling and its downstream pro-metastatic Akt signaling (37, 38). Besides, the activation of RCP by mutp53 also enhances HSP90α secretion, which increases cell motility through interaction with extracellular matrix (ECM) (39). Metabolism reprogramming is also involved in mutp53 induced metastasis. Using a p53^R172H/+^ mice model (R175H in human), Xiong. et al. found that the interaction between mutp53 and ETS2 also induces *Pla2g16* expression, which encodes a phospholipase that catalyzes phosphatidic acid into lysophosphatidic acid and free fatty acid, and both of which have been implicated in promoting migration and metastasis (40–42). Besides, the common polymorphism Pro72Arg at mutp53 enhances migration and metastasis of tumors through its ability to bind and regulate PGC-1α target genes, which is a key regulator in mitochondrial biogenesis and oxidative phosphorylation (43, 44).

### Inducing Chemo- and Radio-Resistance

Chemotherapy and radiotherapy are currently the most widely used therapies for metastatic cancers. However, tumor cells always develop ways to evolve radio- and chemo-resistance capacity to survive these therapies, and mutation in the p53 gene is one of the crucial attempts (45). In this context, mutp53 proteins regulate the expression of several chemo- and radio-resistant genes. MDR1 (multi-drug resistance 1) encodes an energy-dependent efflux pump that mediates the resistance of tumor cells to various hydrophobic cytotoxic drugs (46). Mutp53 proteins strongly upregulate MDR1 expression through ETS1 mediated promoter binding, while the restoration of WT p53 could abolish MDR1 activity by reducing its phosphorylation (47–49). Mutp53 activates the expression of NRF2, which is known to confer both chemo- and radio-resistance (50), including chemo-resistance of cisplatin, apigenin, and radio-resistance of tumor cells (51–53). In triple-negative breast cancer cells, the cooperation between mutp53 and NRF2 was reported to activate proteasome gene transcription, resulting in resistance to proteasome inhibitor carfilzomib (54). Therefore, targeting NRF2 pathway has the potential to increase the curcumin compound induced cell death of mutp53-carrying cancer cells (55). In addition, in cells with WT p53, DNA damage caused by radiotherapy and most chemotherapeutic agents would lead to p53 accumulation and apoptosis. Whereas certain mutp53 has been reported to inhibit caspase-9 and p63/73-dependent induction of *Bax* and *Noxa*, contributing to the anti-apoptotic effects of mutp53 and the insensitivity of mutp53 harboring cells to radio- and chemo-therapies (56–58).

Emerging evidence suggests that the radio- and chemo-resistance capacity are primarily achieved by cancer stem cells (CSC) (59–61). Mutp53 proteins play vital roles in CSC formation and maintenance (62). High prevalence of p53 mutations is reported in poorly differentiated carcinomas and contributes to a stem cell-like transcriptome (63, 64). WT p53 has been reported to repress the expression of several CSC markers including CD44, c-KIT, NANOG and OCT4, while mutations in p53 would lead to loss of repression on these CSC markers, subsequent CSC transformation and the resultant enhanced radio- and chemo-resistance (65).

### Facilitating a Pro-Oncogenic Tumor Microenvironment

It is now accepted that tumor progression and response to therapeutic treatments are not simply dependent on cell autonomous characteristics. The tumor microenvironment consisting mainly of ECM, stromal cells, immune cells, and blood vessels plays a key role in the tumorigenesis and chemoresistance capacity (15). Mutp53 can modulate tumor microenvironment by inducing the secretion of pro-inflammatory cytokines and angiogenesis (66, 67). The p53R248W and D281G mutants can activate the activity of matrix metalloproteinases (MMPs) by repressing the transcription of TIMP3 (68). Consistently, colorectal carcinomas expressing p53^R273H,V216M^ show significant upregulation of MMP9 expression (69). The increased MMP activity results in the degradation of ECM surrounding the tumor cells, leading to enhanced metastasis and invasiveness (68, 70). Mutp53 has also been reported to form complex with HIF1 to upregulate ECM components Viia1 collagen and laminin-γ2 to promote tumor progression (71). In addition, the crosstalk between Mutp53 and the master inflammatory regulator NF-κB pathway has been largely implicated in modulating tumor development and migration (69, 72–74), through the upregulation of a cancer-related gene signature including CXC-chemokines, interleukins (ILs) and ECM-related genes (73–75). Finally, mutp53 was reported to positively regulate the expression of pro-angiogenic factors including IL-8, GRO-α, and VEGF to promote tumor neo-angiogenesis, which is another “hallmark of cancer” (15, 76, 77).

### Therapeutic Strategies for Cancer Harboring p53 Mutations

**Figure 1:**
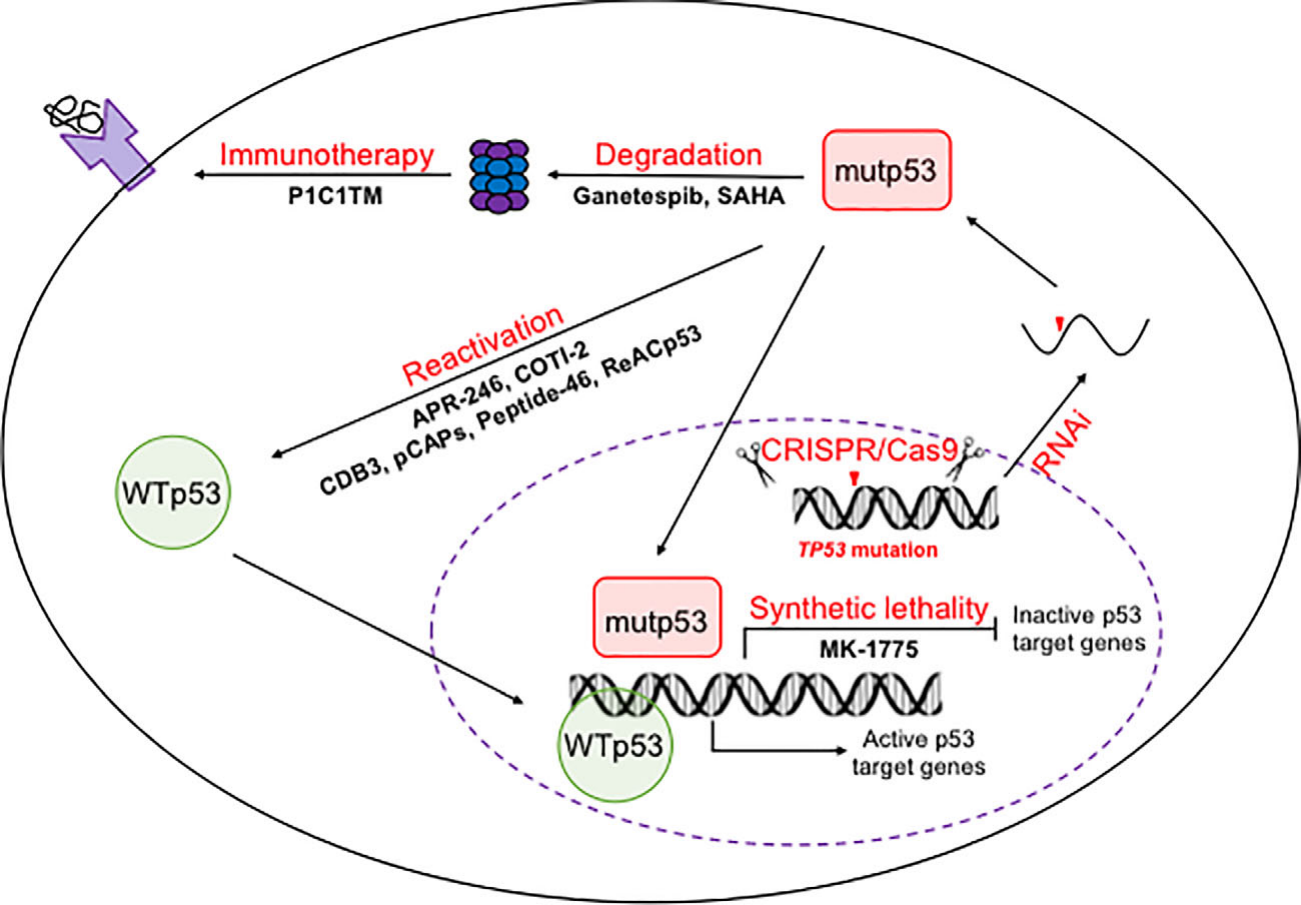
Therapeutic strategies to target p53 mutants. On the DNA level, mutations in *TP53* allele could be reversed back to wild-type ones using CRISPR/Cas9 mediated genome editing. One the mRNA levels, mutp53 mRNA could be silenced by RNAi. On the protein level, mutp53 could be reactivated or trageted for degradation by both small molecule compounds and small peptides. The inability of mutp53 to activate its downstream target genes provides an opportunity for synthetic lethality based therapy. The mutant peptides produced by degradation of mutp53 makes immunotherapies possible.

The reliance of tumors on mutp53 makes it an ideal target for cancer therapy. Therapeutic strategies targeting mutp53 can be divided into three categories, restoring the WT conformation and transcriptional activity of mutp53, targeting mutp53 for degradation, and inducing synthetic lethality (78, 79). To achieve these therapeutic goals, small molecular compounds, synthetic small peptides, CRISPR/Cas9 mediated genome editing, small interference RNAs (RNAi) as well as immunotherapies have been explored (**Figure 1**).

### Small-Molecule-Compounds-Based Therapy Targeting Mutp53

Pharmaceutical targeting of mutp53 is more challenging than targeting oncogenic kinases, which can be easily inactivated by small molecule inhibitors (80). While intensively pursued, no mutp53 targeting regiment has yet been clinically approved. Several promising small molecule drugs under clinical trials are reviewed below (ClinicalTrials.gov). Some other drugs that have achieved promising results in preclinical studies have been extensively reviewed elsewhere (79, 80).

#### APR-246

APR-246 is a methylated analogue of PRIMA-1, which was identified as a low-molecular-weight compound that restores wildtype function of mutp53 (81, 82). While methylene quinuclidinone (MQ) is the common bioactive decomposition product of both APR-246 and PRIMA-1, the bioactivity of APR-246 is much higher, possibly due to its higher lipophilicity and cell permeability (80, 82). The covalent binding of MQ to p53 core domain, primarily *via* cysteines 124 and 277, enhances the thermostability of mutp53 and contributes to the refolding of mutp53 to WT conformation, thus enables the re-induction of p53 target genes such as *CDKN1A* (83–85). Numerous preclinical studies using rodent models have revealed the tumor suppressive effect of APR-246 on mutp53-expressing tumor cells of various origins (86–90). Furthermore, the phase I study has verified that APR-246 is safe at predicted therapeutic plasma levels with a favorable pharmacokinetic profile, and most importantly, can induce considerable p53-dependent biological effects in cancer patients with p53 mutations (91, 92). Therefore, APR-246 is considered as a promising first-in-class mutp53-targeting drug. Now, several phase II clinical trials of APR-246 are ongoing, including a systemic carboplatin combination chemotherapy with APR-246 in patients with platinum sensitive recurrent high grade serous ovarian cancer with mutated p53 (NCT02098343), a combination of APR-246 with azacytidine in p53 mutant myeloid neoplasms (NCT03072043) and a combination of APR-246 with 5-FU and cisplatin in oesophageal cancer (NCT02999893).

#### COTI-2

COTI-2 is a novel thiosemicarbazone derivative that is active against multiple human cancers from different origins (93). The anti-tumor activity of COTI-2 is at least partially achieved by promoting the refolding and therefore the DNA binding capacity of mutp53, leading to the reactivation of wildtype p53 target genes including *CDKN1A*, *PUMA*, and *NOXA*. Besides, MAPK and mTOR pathways are also involved in COTI-2 induced apoptosis or senescence (80, 94, 95). COTI-2 is effective at nanomolar concentrations *in vitro*, and is proved to be safe and well-tolerated in xenograft mouse models (93). Following studies revealed that COTI-2 was synergistic in combinations with cytotoxic chemotherapeutics without exerting significant toxicities *in vivo*. In addition, tumor cells resistant to chemotherapeutic agents exhibit no or little cross-resistance to COTI-2, highlighted the potential of COTI-2 in salvage treatment after current first- and second-line treatment failures (96). Based on these observations, a phase I trial of COTI-2 as monotherapy or combination therapy in gynecological tumors and head and neck squamous cell carcinoma (HNSCC) with confirmed p53 mutations is currently being performed (NCT02433626).

#### Ganetespib

In contrast to the relative low levels of wildtype p53 in unstressed physiological conditions, mutp53 is in most cases highly expressed in tumor cell, which is achieved by its cooperation with HSP90 chaperone machinery that inhibit the activity of its primary E3 ubiquitin ligase MDM2 and CHIP (97, 98). This hyperstabilization of mutp53 largely contributes to its dominant-negative and oncogenic GOF activities, and is the foundation of anti-tumor therapies aimed to induce mutp53 degradation. Ganetespib is a highly efficient HSP90 inhibitor (99), which is 50-fold more potent than the first-generation HSP90 inhibitor 17AAG in degrading mutp53 and killing mutp53 cancer cells (100). *In vivo* studies suggested that ganetespib extended the survival of tumor-bearing R172H (corresponding to R175H in human) and R248Q *Trp53* knock-in mice, while have no effect on their corresponding *Trp53^-/-^* littermates (100). Meanwhile, in *Trp53^R248Q/-^* mice bearing T-lymphomas, ganetespib synergizes with cyclophosphamide to suppress tumor growth and extend survival (101). However, it is worth noticing that mutp53 is not the only target of HSP90, instead, HSP90 regulates the activation and stability of a diverse array of oncogenic proteins including HER2, mutant EGFR, and mutant BRAF (99). Even though phase II clinical trials in metastatic breast cancer, malignant peripheral nerve sheath tumors and advanced non-small cell lung cancer all reported that the effect of ganetespib alone or in combination with other anti-tumor drugs did not meet the criteria for overall response rate, subgroups of these patients showed positive responses, which might be attributed to their specific genetic background (102–104). Therefore, more extensive clinical trials with ganetespib are needed.

#### SAHA

Histone deacetylase (HDAC) inhibitors are another group of compounds that are widely reported to reduce the levels of mutp53. SAHA (suberoylanilide hydroxamic acid) is a FDA-approved HDAC inhibitor for the treatment of T cell lymphomas (105). Recent studies found that SAHA exhibits preferential cytotoxicity for mutp53, rather than WT and null p53 cancer cells in certain kinds of human cancers, and also strongly sensitizes mutp53 harboring cancer cells to chemotherapies (100, 106, 107). Mechanistically, SAHA could destabilize mutp53 through inhibition of the HDAC6-HSP90 chaperone axis, and at the same time, inhibit the transcription of mutp53 through HDAC8 (106–109).

#### MK-1775

p53 is mainly responsible for the G_1_/S cell cycle arrest, while in mutp53 harboring cancer cells, the abrogation of this checkpoint results in direct S phase entry even in the presence of DNA damage, making the cells more dependent on G_2_/M checkpoint to maintain genomic stability (110). In this context, further inactivation of G_2_/S checkpoint will lead to unscheduled mitotic entry of cells with extensive DNA damage, resulting in mitotic catastrophe (111, 112). This synthetic lethality provides an ideal opportunity for therapeutic targeting of mutp53 harboring cancer cells. Wee-1 is a tyrosine kinase that involved in DNA damage induced G_2_/M cell cycle arrest by inhibiting CDK1 activity (113). Its specific inhibitor MK-1775, therefore, was reported to show amplified anti-tumor activity specifically in p53 mutant cancer cells. MK-1775 significantly elevated the efficacy of cisplatin, vorinostat (HADC inhibitor), or alisertib (aurora kinase A inhibitor) in HNSCC cells expressing high-risk mutp53 both *in vitro* and *in vivo*, while tumor cells bearing wildtype p53 displayed minimal response to MK-1775 (114–117). Consistently, MK-1775 was also reported to sensitize p53 mutant colon cancer cells to the DNA damage associated drug irinotecan (118). Currently, a randomized phase II study evaluating MK-1775 in combination with paclitaxel and carboplatin in adult patients with platinum sensitive p53 mutant ovarian cancer is ongoing (NCT01357161).

### Genetic Approach to Target Mutp53

#### CRISPR/Cas9 and RNAi

CRISPR/Cas9-based genome editing appears to be a straight-forward therapeutic strategies for tumor cells expressing p53 mutants. By directly replacing the TP53 414delC frameshift mutation locus with a functional copy, Batir et al. successfully restored the wild-type *TP53* genotype and phenotype in prostate cancer cells (119). CRISPR/Cas9 has also also employed in a p53 genetic sensor system which specifically and efficiently killed p53-deficient cancer cells (120). However, the high risk of genome instabiliy induced by CRISPR/Cas9 should be rigorously considered (121, 122). Small interference RNAs could specifically eliminate mutant p53 mRNA without affecting the wild-type one, However, the specificity and *in vivo* efficacy of such RNAi remains to be elucidated.

#### Small Peptides

The goal of small peptide based therapies is to restore wild-type p53 function, either by restabilization of mutp53 or inhibition of the aggregation of mutp53. The denaturation of mutp53 at physiological temperature largely contributes to the inabilily of mutp53 to activate downstream tumor suppressive genes. Therefore, several mutp53 reactivating peptides, such as CDB3, peptide-46 and pCAPs, have been identified to restore wildtype p53 activities to cancer cells (123, 124). On the other hand, a large portion of p53 mutants have been reported to form protein aggregates, which contributes to the GOF properties that promote tumor growth. In this context, ReACp53, a cell-penetrating peptide inhibitor of mutp53’s aggregation, which resembles the transactivation inhibitory domain of p63, showed promising anti-cancer effect in both ovarain and prostate cancer models *in vivo* (125–127).

#### Immunotherapy

While the accumulated mutp53 escapes from MDM2-mediated degradation, it can still be degraded in a MDM2-independent and proteasome-dependent pathway, generating peptides that are eventually presentated on tumor cell surface by class I molecules of the major histocompatibility comples (MHC). Therefore, mutp53 and the p53-derived mutant peptide-MHCs could serve as potental therapeutic targets for immunotherapies (128, 129). Even though peptides containing mutp53 sequences are rare due to MHC-binding restrictions, an engineered T cell receptor-like (TCRL) antibody P1C1TM, which is specific for a wild-type p53_125-134_ peptide presented by the HLA-A24:02 (HLA-24) MHC allele, was reported to be able to discriminate between mutant and wild-type p53-expressing HLA-A24^+^ cells based on antigen expression levels. This elegant interaction between intracellular mutp53 and targetable cell surface peptide-MHC complex enables efficient antibody dependent cellular cytotoxicity of mutp53 expressing cells both *in vitro* and *in vivo* (129). In the future, it is worthwhile to identify new cell surface peptides specifically derived from mutp53.

## Conclusion

The addiction of cancer cells to mutp53 makes it an attractive target for cancer therapy. By elucidating the mechanisms of GOFs of mutp53, numerous strategies have been explored to specifically target mutp53. One highly pursued strategy is to develop small molecule compound and small peptide to restore the conformation and transcriptional activity of wild-type p53 to the mutp53. This strategy is challenging due to the relative undruggable nature of mutp53 with various thermostability or conformational structures. Therefore, high-resolution structural and functional analysis of the full length WT and mutp53 will be required to design more effective small molecule compounds and small peptides to target mutp53. However, it is noteworthy that our group recently found that hepatocellular carcinomas (HCCs) often retain the wild-type p53 to suppress oxidative phosphorylation and increase glycolysis, thereby promoting HCC progression (130). In this context, strategies aiming to restore WT p53 activities of mutp53 might instead promote tumorigenesis under certain circumstances, therefore requires rigorous validation before clinical trials. Synthetic lethality, gene editing, siRNA silencing, and immunotherapy are promising strategies to target mutp53 to treat mutp53-expressing tumors, however, these approaches all have intrinsic problems that must be optimized before clinical applications. In this context, future effort should be devoted to improve the specificity, efficacy, and safety of these promising strategies to target mutp53-expressing human cancers.

## Author Contributions

GZ, CP, JB, BL, CL, YX, and XF drafted the manuscript. All authors contributed to the article and approved the submitted version.

## Funding

This work was supported by the National Natural Science Foundation of China (Nos. 91959204, 81930084, 815300045, 81871197, 81703092), the leading talents of Guangdong Province Program (No. 00201516), Shenzhen “Sanming” Project of Medicine (SZSM201602102), and the Development and Reform Commission of Shenzhen Municipality (S2016004730009).

## Conflict of Interest

The authors declare that the research was conducted in the absence of any commercial or financial relationships that could be construed as a potential conflict of interest.
